# Solvent-Free Injection in Supercritical Fluid Chromatography Using Sintered Glass Deposition

**DOI:** 10.6028/jres.093.158

**Published:** 1988-12-01

**Authors:** Thomas J. Bruno

**Affiliations:** National Institute of Standards and Technology Boulder, CO 80303

**Keywords:** chromatography, injection, supercritical fluid chromatography, supercritical fluids

## Abstract

Sample injection in supercritical fluid chromatography (SFC) is usually performed using a combination of apparatus from liquid chromatography and capillary gas chromatography. The device most often consists of an injection valve (of the type used in liquid chromatography) followed by a flow splitter controlled by a restrictor. It is sometimes desirable to inject samples in the absence of a solvent, as in physicochemical applications of SFC. In this article, two simple modifications to a conventional sampling valve system are presented which allow solvent-free injection. Using these devices, sample (in a solvent) is deposited on a sintered glass bed. After removal of the solvent by mild heating and evacuation, the sample loop is filled with the supercritical carrier and the valve is switched to the inject position.

Sample introduction in analytical applications of supercritical fluid chromatography (SFC) is usually performed using a high-pressure multiport sampling valve (of the type used for high-performance liquid chromatography–HPLC) equipped with a flow splitter. The splitter is most often of fixed split ratio (for a given pressure and temperature): the ratio is determined and controlled by a fused silica capillary restrictor. The sample, which is usually a solid or liquid dissolved in an appropriate solvent, is syringe loaded into the sample loop of the multiport valve, and injection is achieved by switching the position of the valve. The solvent will be separated from the sample species by the physical and chemical interactions with the stationary phase in the column.

In some nonanalytical applications of supercritical fluid chromatography it is often desirable to inject a sample (which may be of low volatility) in the absence of solvent. Such applications are typically in physicochemical measurement studies. As an example, we may consider the chromatographic determination of binary diffusion coefficients using the Taylor-Aris method [1–5]. In this technique, a sample is injected onto the head of an open tubular column as a sharply defined spike. As the carrier moves the sample through the length of the tube, the initial sharply defined spike will broaden into a Gaussian-like profile. For this experiment, the column is uncoated and ideally should be inert, and the presence of a solvent would ruin the measurement.

In order to allow convenient injection of relatively nonvolatile solutes for this type of measurement, we have used two approaches to provide a solvent-free injection. In the first approach, a standard multiport injection valve has been modified to accept the deposition of sample on a small section of sintered glass placed within the sample loop. In the second approach, an extractor based on sintered glass deposition precedes the sampling valve. Sintered glass was chosen as the deposition material for several reasons. First, since glass has a relatively high energy surface, one would expect a conveniently-sized aliquot of sample to be easily loaded. In addition, there is no danger of the sintered glass bed being displaced from its position in the loop or extractor. An initial series of devices packed with small beds of chromatographic supports (such as Tenax-GC^1^) was unsatisfactory for this reason.

In the first approach, the sample loop is fitted with a nichrome heater wire, and the valve block with a cartridge heater to provide temperature control. A schematic diagram of the modified sample loop is provided in figure 1. The sintered glass, which was obtained from a commercial supplier as a 0.16-cm (0.0625-in) thick disk, was cut into cylindrical sections (of 0.16-cm diameter) using a core drill. To fabricate a loop, one or more of these wafers are interposed in the center of a sample loop [consisting of 316 stainless steel tubing of 0.02-cm (0.009-in) inside diameter and 0.16-cm (0.0625-in) outside diameter] constrained by a small stainless steel cylinder [0.32-cm (0.125-in) outside diameter and 0.16-cm (0.0625-in) inside diameter]. This outer cylinder is brazed to the sample loop using a hydrogen torch. A hydrogen torch was employed to avoid using flux, which could contaminate the sintered glass bed. The sampling loop thus modified is then installed as part of a high-pressure analogue of a sampling system described previously [6]. This sampling system contains provisions for pressurization, venting, and evacuation of the sample loop. The high-pressure limit of this sample loop is constrained by that of the sampling valve [approximately 41 MPa (6000 psi)]. Calculations indicate, however, that the maximum allowable working pressure of the modified loop itself is in excess of 96 MPa (approximately 14,000 psi).

The use of this sampling device is quite simple. A solvent-borne solute is loaded via syringe into the sampling loop as one would in HPLC. In this case, the more volatile the solvent and the less volatile the solute, the better. The sample loop and valve are then warmed and the solvent vapors vented. The loop is then evacuated to remove as much residual solvent vapor as possible. Naturally, the longer the evacuation time, the more solvent will be removed. A heating-evacuation cycle of between 5 and 7 min is usually sufficient to remove all but trace quantities of most common solvents.

The chromatogram presented in figure 2 was obtained using this sampling technique in a developmental supercritical fluid chromatograph designed and built for physicochemical measurements. The sample in this case was a 0.01 percent (mass/mass) solution of naphthalene in methylene chloride. The solution was loaded into the loop using an HPLC syringe, and the methylene chloride solvent was removed by a heating-evacuation cycle (at a temperature of approximately 50 °C for 5 min). The loop was then pressurized with carbon dioxide at 13.8 MPa (2000 psi), with the loop and sampling valve being held at 50°C. An equilibration time of approximately 1 min was allowed for the dissolution of the naphthalene in the carbon dioxide. Switching of the sampling valve then resulted in the injection into the carrier stream at a pressure 20.7 MPa (3000 psi) and 130°C (the conditions of the chromatographic experiment). The pressurization step described above is done at a pressure lower than the column initial pressure so as to counteract somewhat the deleterious effect of the relatively large volume of the modified loop. In the case of figure 2, the column was a 3050-cm uncoated stainless steel tube [0.23-cm (0.009-in) inside diameter], and detection was done using a modified flame ionization detector [7]. The symmetry of the peak is indicative of negligible adsorptive interference by the sintered glass bed.

There are a number of advantages associated with this method of injection. The first is, of course, the removal of the solvent when solvent-free injection is required. In addition, there is a greatly reduced need to use a flow splitter since most of the applied sample is solvent, which is removed during the heating-evacuation step. To reduce the amount of solute injected, one simply dilutes the solution applied to the sintered glass. The solute is then actually injected in a solution of the supercritical carrier. The major disadvantage experienced with the technique is the relatively long time involved in completing an injection (approximately 8 min). Improvements along these lines are currently being pursued. Since another disadvantage is the relatively large loop volume, attempts are being made to minimize the size of the sintered glass bed. The problems caused by large loop volume (such as peak broadening and pressure pulsation) appear to be relatively minor, however, as judged from comparative HETP (height equivalent to a theoretical plate) determinations.

A second approach we have had success with makes use of a small extractor placed ahead of the sampling valve. This approach is similar to that taken by others in physicochemical measurements [8,9]. The extractor is shown schematically in figure 3. The manifold arrangement used with this extractor is shown in figure 4. The extractor itself was machined from a section of 316 stainless steel bar, 12.7 cm in diameter and 4 cm in length. A hole (0.32-cm diameter) bored through the center of the bar accommodates a section of cut sintered glass, as in the sample loop approach described earlier. The extractor is sealed at either end using threaded or brazed fittings. A syringe loading tube is brazed onto the side of the extractor body, and is capped using a compression fitting plug.

To use this extractor, a solvent-borne sample is loaded onto the sintered glass bed through the loading tube using a conveniently-sized syringe. After capping the loading tube with a pressure fitting (not shown in fig. 3), the extractor and transfer lines are evacuated using the vacuum valve. Solvent evaporation is aided by raising the temperature of the entire manifold, which is contained in an air bath. The extractor is then filled with the carrier using the inlet valve. As can be seen from figure 4, the position of the chromatographic injection valve determines if the loop is filled. Usually, the injection valve is in the inject or flow position until carrier and sample have had time to equilibrate. After the sampling valve is switched to the inject position, the inlet valve may be reopened to insure that the carrier pressure will be the same in both the chromatographic column and the sampling loop. The density will also depend upon the temperature, thus to achieve density equivalence, the chromatographic column and injection manifold must be at the same temperature. This generally will pose no difficulty, since one sample loading can usually provide enough solute for dozens of injections.

## Figures and Tables

**Figure 1:**
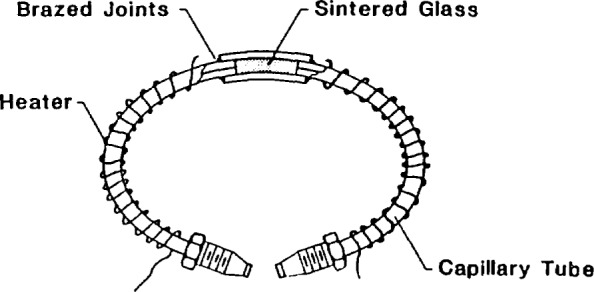
A schematic diagram of the modified sample loop containing a cylinder of sintered glass.

**Figure 2:**
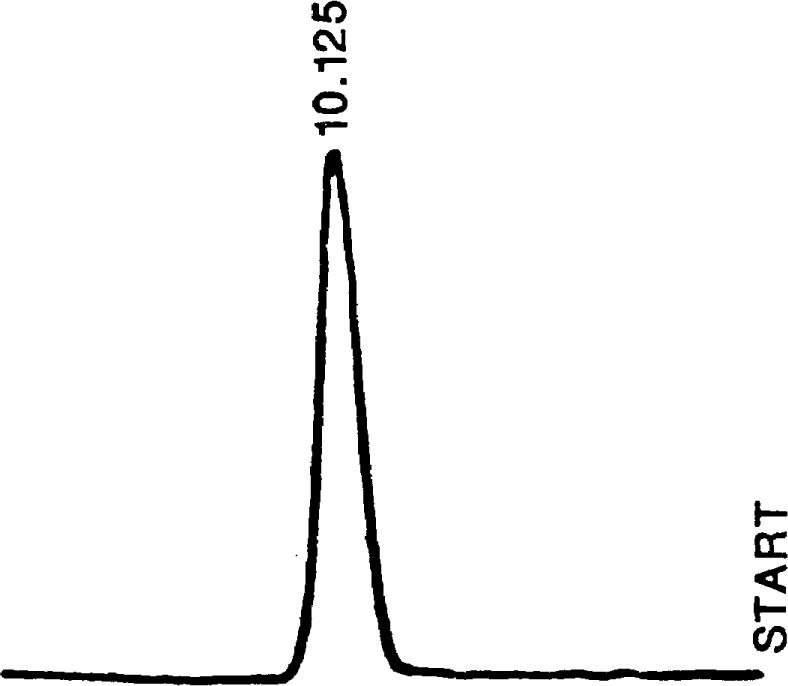
A chromatogram showing the FID response of naphthalene in supercritical carbon dioxide at 20.7 MPa (3000 psi) and 130°C. The electrometer was set at a range of 10^−11^, with attenuation of 1.

**Figure 3:**
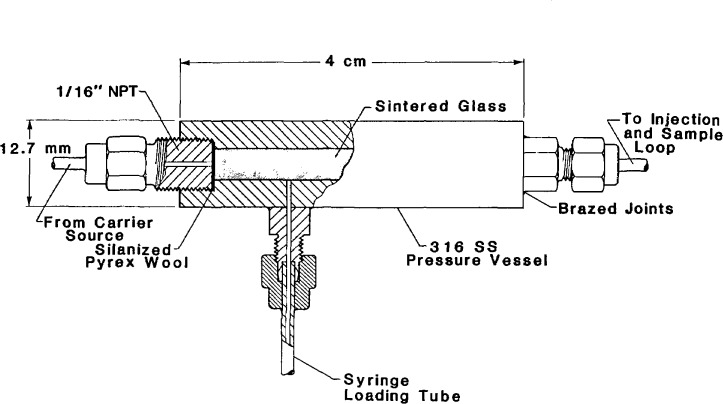
A schematic diagram of the extractor (which precedes the sampling valve). The manifold for this extractor is shown in figure 4.

**Figure 4:**
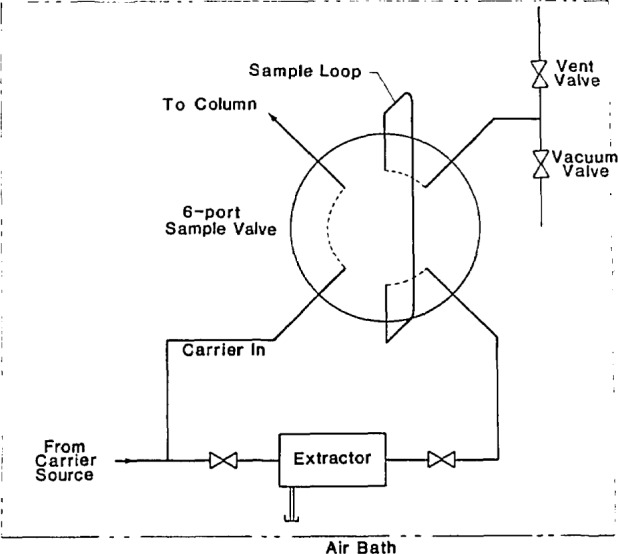
A schematic diagram of the manifold which controls the extractor (which is shown in fig. 3). The sampling valve is shown in the “fill” position.

## Footnotes

1 Certain commercial equipment, instruments, or materials are identified in this paper in order to adequately specify the experimental procedure. Such identification does not imply recommendation or endorsement by the National Institute of Standards and Technology, nor does it imply that the materials or equipment identified are necessarily the best available for the purpose.
